# Dynamic causal modelling revisited

**DOI:** 10.1016/j.neuroimage.2017.02.045

**Published:** 2019-10-01

**Authors:** K.J. Friston, Katrin H. Preller, Chris Mathys, Hayriye Cagnan, Jakob Heinzle, Adeel Razi, Peter Zeidman

**Affiliations:** a The Wellcome Trust Centre for Neuroimaging, University College London, United Kingdom; b Neuropsychopharmacology and Brain Imaging, Department of Psychiatry, Psychotherapy and Psychosomatics, University Hospital for Psychiatry Zurich, 8032 Zurich, Switzerland; c Max Planck UCL Centre for Computational Psychiatry and Ageing Research, University College London, United Kingdom; d MRC Brain Network Dynamics Unit (BNDU), Department of Pharmacology and Nuffield Department of Clinical Neurosciences, University of Oxford, Oxford, UK; e Translational Neuromodeling Unit, Institute for Biomedical Engineering, University of Zurich and ETH Zurich, 8032 Zürich, Switzerland; f Department of Electronic Engineering, NED University of Engineering and Technology, Karachi, Pakistan

**Keywords:** Dynamic causal modelling, Haemodynamic models, Neural mass models, Effective connectivity, Bayesian

## Abstract

This paper revisits the dynamic causal modelling of fMRI timeseries by replacing the usual (Taylor) approximation to neuronal dynamics with a neural mass model of the canonical microcircuit. This provides a generative or dynamic causal model of laminar specific responses that can generate haemodynamic and electrophysiological measurements. In principle, this allows the fusion of haemodynamic and (event related or induced) electrophysiological responses. Furthermore, it enables Bayesian model comparison of competing hypotheses about physiologically plausible synaptic effects; for example, does attentional modulation act on superficial or deep pyramidal cells – or both? In this technical note, we describe the resulting dynamic causal model and provide an illustrative application to the attention to visual motion dataset used in previous papers. Our focus here is on how to answer long-standing questions in fMRI; for example, do haemodynamic responses reflect extrinsic (afferent) input from distant cortical regions, or do they reflect intrinsic (recurrent) neuronal activity? To what extent do inhibitory interneurons contribute to neurovascular coupling? What is the relationship between haemodynamic responses and the frequency of induced neuronal activity? This paper does not pretend to answer these questions; rather it shows how they can be addressed using neural mass models of fMRI timeseries.

## Highlights

• This paper describes a DCM for fMRI based on neural mass models and canonical microcircuits.
• This enables the (Bayesian) fusion of EEG and fMRI data.
• That encompasses the formal modelling of neurovascular coupling.
• Offers a surprising insight into the relationship between haemodynamic and electrophysiological responses.

## Introduction

Over the past decade, dynamic causal modelling (DCM) has become the predominant way of characterising effective connectivity within networks of distributed neuronal responses (Daunizeau et al., 2011, Razi and Friston, 2016), as measured with fMRI (Friston et al., 2003) or electromagnetic responses (David et al., 2006). Although there have been technical advances in the dynamic causal modelling of the fMRI timeseries; such as the introduction of stochastic and spectral DCM (Li et al., 2011, Friston et al., 2014b) – and innovations such as Bayesian model reduction and hierarchical (empirical) models for group studies (Friston et al., 2015, Friston et al., 2016) – the underlying model of neuronal and haemodynamics has remained basically unchanged (Stephan et al., 2007, Marreiros et al., 2008, Stephan et al., 2008). This model is based upon a second-order Taylor approximation to neuronal activity, as described with equations of motion. In parallel, there have been rapid developments in the dynamic causal modelling of electrophysiological timeseries; both in terms of event related and induced (cross spectral) responses (Moran et al., 2011, Friston et al., 2012). This branch of dynamic causal modelling has addressed more physiologically informed questions about the role of forward and backward connections in cortical hierarchies and how experimental effects are mediated at the synaptic level: e.g., Boly et al., 2011, Auksztulewicz and Friston, 2015, Bastos et al., 2015. In this paper, we combine these two strands of modelling to provide a DCM for fMRI timeseries based on generative or forward models used in DCM for electromagnetic timeseries. This is effected by replacing the Taylor approximation used in DCM for fMRI with the differential equations used in DCM for EEG and MEG. These differential equations are based upon standard neural mass models of neuronal dynamics within a canonical microcircuit (Douglas and Martin, 1991, Jansen and Rit, 1995, Bastos et al., 2015). This combination offers several advantages:

First, it allows one to specify hypotheses or models of distributed responses – as measured with fMRI – that are more physiologically grounded. For example, one can specify hierarchal architectures with distinct (laminar-specific) forward and backward connections. Furthermore, one can specify experimental effects in terms of changes in either extrinsic (between-region) or intrinsic (within-region) connectivity at the level of specific neuronal populations; e.g., superficial or deep pyramidal cells (Brown and Friston, 2012, Auksztulewicz and Friston, 2015).

Second, it provides a framework within which to combine different modalities. Crucially, because the generative model used for MRI can also generate evoked or induced electromagnetic responses, one can use both modalities to inform the parameters of the same model. Furthermore, the form of fusion afforded by using the same (neuronal) model frees one from the tyranny of having to acquire fMRI and electrophysiological data concurrently. In other words, one can first analyse EEG data using event related or induced responses to estimate the connectivity and synaptic parameters of a DCM. These posterior estimates then become prior probability distributions for a subsequent inversion, using fMRI data, to estimate regionally specific haemodynamic parameters. This is known as *Bayesian belief updating* and properly accounts for the conditional dependencies between neuronal and haemodynamic parameters. The resulting multimodal *Bayesian fusion* provides a comprehensive characterisation of functional anatomy that exploits the temporal (electromagnetic) and spatial (fMRI) nature of different imaging modalities. We will illustrate multimodal fusion in a subsequent paper. In this paper, we consider the form of the DCM and provide some illustrative applications to show how far one can get using fMRI data alone.

Third, having a physiologically informed neuronal and haemodynamic model means that one can, in principle, resolve outstanding questions about the nature of the BOLD response. For example, does the BOLD response reflect afferent presynaptic activity from distant (extrinsic) sources or does it report local activity mediated by recurrent (intrinsic) connectivity (Attwell and Iadecola, 2002, Logothetis, 2008)? To what extent do inhibitory interneurons contribute to BOLD signals (Arthurs and Boniface, 2002, Kann et al., 2014)? Is the BOLD signal generated in superficial cortical layers, deep cortical layers or both (Goense et al., 2012, Poplawsky et al., 2015)? And what are the haemodynamic correlates of event-related desynchronisation and fast (oscillatory) activity (Singh et al., 2002; Laufs et al., 2003; Kilner et al., 2005; Murta et al., 2015; Scheeringa et al., 2016)?

Our main aim in this paper is to show how such questions could be answered using the framework introduced below, rather than providing definitive answers to the aforementioned questions. Our illustrations are therefore restricted to the analysis of a single time series from a single subject (Buchel and Friston, 1997). These data are the same time series that have been used to illustrate previous developments in dynamic causal modelling and are available from the SPM website (http://www.fil.ion.ucl.ac.uk/spm/).

This paper comprises four sections. The first introduces the neural mass model that constitutes the neuronal part of the DCM for fMRI. The second section illustrates its application to empirical data acquired during an ‘attention to visual motion’ paradigm. This section illustrates how biological questions about the synaptic mediation of attention can be posed at the level of specific neuronal populations; such as the contribution of superficial versus deep pyramidal cells to attentional effects in observed data. The third section turns to questions about haemodynamics; for instance whether BOLD responses are driven by extrinsic or intrinsic presynaptic activity – and whether inhibitory interneurons have a role to play. The final section considers the multimodal capabilities of the model by simulating the induced responses that would have been seen using local field potentials, based upon the parameters estimated from the fMRI data. This section focuses on the relationship between desynchronisation (and the expression of gamma activity) and BOLD responses disclosed by experimental changes in attention set. We conclude with a brief discussion of conclusions that can be drawn from this sort of modelling.

## Dynamic causal modelling with neural mass models

This section reviews the structure of the canonical microcircuit DCM. The requisite equations can be found in the figures, while a glossary of variables and mathematical expressions can be found in accompanying tables. Dynamic causal modelling refers to the inversion of generative or forward (state-space) models of observable responses, given a model of how neuronal activity causes measurements (e.g. fMRI, EEG, MEG timeseries). This inversion generally uses standard Variational Laplace (Friston et al., 2007) procedures to estimate model parameters and their marginal likelihood or *model evidence* for inferences about specific connections (Daunizeau et al., 2011) and network architecture respectively (Penny et al., 2004). The latitude of inferences therefore rests on the nature of the models used to explain the data.

Usually, dynamic causal models have a neuronal part that describes distributed neuronal activity with a greater or lesser degree of biological realism and a measurement part that converts neuronal activity into measurable observations. For fMRI, this involves specifying haemodynamic models, while for electromagnetic data it usually reduces to specifying a lead field or electrode gain. The separation into neuronal and measurement models is important, because it allows the same neuronal model to predict multiple modalities, using different measurement models. This means one can use multimodal data features to estimate the model's evidence and parameters. In this paper, the neuronal model is exactly the same model used in DCM for EEG (and MEG), while the haemodynamic model is exactly the same as has been used for fMRI over the past decade (Stephan et al., 2007).

There have been several advances in haemodynamic modelling that have been informed by different sorts of (e.g., arterial spin labelling) fMRI data (Havlicek et al., 2015) – and the modelling of laminar specific responses measured with high-resolution fMRI (Heinzle et al., 2016). However, here, we will use the original haemodynamic model for simplicity and consistency with previous work; noting that it is easy to incorporate more advanced haemodynamic models and evaluate the improvement using Bayesian model comparison (Stephan et al., 2007). A potentially important issue here is whether the haemodynamic model predicts laminar specific fMRI signals. In this paper, we are not concerned with high-resolution fMRI and therefore model the fMRI signal as a mixture of contributions from different cortical layers, modelling the relative contributions with unknown neurovascular coupling parameters. This should be contrasted with alternative strategies that include spatial aspects within the haemodynamic model that would be necessary for data that is spatially resolved at the (sub) millimetre level (Heinzle et al., 2016, Puckett et al., 2016). We will first describe the DCM for a single cortical region or source (i.e., node) and then consider distributed networks of sources (i.e., graphs) and their extrinsic connections.

### Dynamic causal models with four neuronal populations per region

**Fig. 1:**
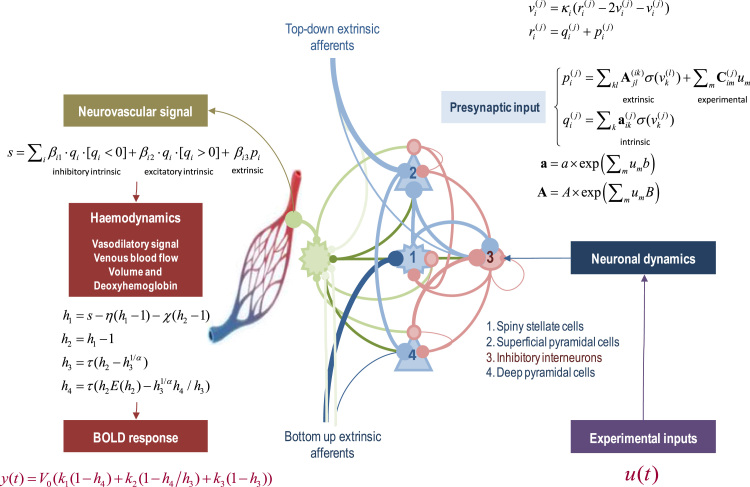
Schematic summarising the generative model for each region or node. This model comprises two sets of differential equations modelling neuronal dynamics and haemodynamics respectively. These are coupled via a linear (neurovascular) mapping, such that the neuronal states provide an input to the haemodynamics. Experimental inputs perturb neuronal dynamics that are modelled with a canonical microcircuit. This microcircuit comprises four neuronal populations, comprising spiny stellate cells, superficial pyramidal cells, inhibitory interneurons and deep pyramidal cells. Each population is equipped with two hidden states whose dynamics are described by the second-order ordinary differential equation in the figure. These equations of motion model the depolarisation of each population in response to experimental inputs and afferents from other populations in the same (intrinsic) and other (extrinsic) nodes. The four populations are coupled via intrinsic connections that correspond to known inter-and intralaminar connectivity. Pre-synaptic activity at each subpopulation is then used to drive haemodynamic responses, through local collaterals innervating astrocytes, whose (endfeet) processes release vasodilatory signals. These signals then enter a standard haemodynamic model to generate a BOLD signal. In this graphic, pink connections are inhibitory, blue connections are excitatory and green connections correspond to collateral projections mediating neurovascular responses. Please see Table 1, Table 2 for a list of the variables (and their prior densities). The square brackets are Iverson brackets, returning one when the expression is true and zero otherwise.

Fig. 1 summarises the generative model for each region or node. This model comprises two sets of differential equations modelling neuronal dynamics and haemodynamics respectively. These are coupled via a linear (neurovascular) mapping, such that neuronal states provide input to the haemodynamics. Experimental inputs (e.g., visual input from the lateral geniculate) perturb neuronal dynamics that are modelled with a canonical microcircuit. This microcircuit comprises four neuronal populations (per node), corresponding to spiny stellate cells, superficial pyramidal cells, inhibitory interneurons and deep pyramidal cells. These are denoted by populations 1–4. The three excitatory populations model the granular, supragranular and infragranular excitatory cells, while inhibitory interneurons neurons are treated as a single inhibitory population. Each population is equipped with two implicit hidden states, whose dynamics are described by the second-order ordinary differential equation in the figure. These model the responses of each neuronal population to presynaptic (firing rate) inputs that include experimental inputs (mediated by $C_{im}^{\left(j\right)}$), extrinsic afferents from other regions (mediated by extrinsic connectivity $A_{jl}^{\left(ik\right)}$), and intrinsic afferents from other neuronal populations (mediated by intrinsic connectivity $a_{ik}^{\left(j\right)}$). This class of neural mass model is known as a *convolution* model (Moran et al., 2013) and, effectively, convolves presynaptic input with a synaptic kernel to provide a depolarizing or hyperpolarizing input to postsynaptic populations via intrinsic and extrinsic connections. The connectivity strengths are free parameters of the model (see Table 1), whereas the population-specific time constants $\kappa_{i}$ are fixed – when modelling fMRI data – to ensure efficient model inversion.

**Table 1:** Parameters of a neuronal model (see also Fig. 2).

	**Description**	**Parameterisation**	**Prior**
$\kappa_{i}$	Postsynaptic rate constant of the *i*-th neuronal population in each of *N* regions	$\begin{array}{c} \exp(\theta_{\kappa })\cdot \kappa_{i}\\ \kappa =[256,128,16,32] \end{array}$	$p(\theta_{\kappa })=N(0,0)$
$a_{ik}^{\left(j\right)}$	Intrinsic connectivity to population *i* from population *k* in each region *j*	$\exp(\theta_{a})\cdot a$	$p(\theta_{a})=N(0,0)$
$b_{ikm}^{\left(j\right)}$	Change in intrinsic connectivity caused by the *m-*th input in region *j*	$\theta_{b}\in {\mathbb{R}}^{4\times 4\times J\times M}$	$p(\theta_{b})=N(0,\frac{1}{8})$
$A_{jl}^{\left(ik\right)}$	Extrinsic connectivity to population *i* in region *j* from population *k* in region *l*	$\exp(\theta_{A})\cdot A$	$p(\theta_{A})=N(0,\frac{1}{8})$
$B_{jlm}^{\left(ik\right)}$	Change in extrinsic connectivity caused by the *m-*th input	$\theta_{B}\in {\mathbb{R}}^{4\times 4\times N\times N\times M}$	$p(\theta_{B})=N(0,\frac{1}{8})$
$C_{im}^{\left(j\right)}$	Direct driving effect of the *m-*th input on population *i* in region *j*	$\theta_{C}\in {\mathbb{R}}^{4\times N\times M}$	$p(\theta_{C})=N(0,\frac{1}{32})$

For simplicity, conduction delays between neuronal populations have been omitted from the differential equations in Fig. 1. These delays have prior expectations of 1 millisecond for intrinsic connections and 16 milliseconds for extrinsic connections. Usually, when fitting electromagnetic data, conduction delays are optimised during model inversion and can have an important effect on neuronal responses. They are therefore included in the neural mass model for fMRI (but are fixed at their prior mean). In principle, one could use fMRI data to estimate conduction delays on a millisecond timescale. This is because changes in conduction delays can have profound effects on the amplitude of measurable responses, both in terms of induced responses in electromagnetic studies and haemodynamic responses in fMRI. This fact allows us to address an occasional misconception about DCM: the important information that enables parameter estimation is not contained in the timing of haemodynamic responses – it is the subtle but systematic changes in the amplitude of distributed regional responses that enables efficient parameter estimation. It may seem odd to suggest that the amplitude of BOLD signals contains information about neuronal dynamics at the millisecond timescale; however, this assertion becomes less mysterious when appreciating that one can measure velocity using the colour of light (i.e., the Doppler Effect). The systematic relationship between axonal conduction delays (respectively, velocity) and the pattern of fMRI responses (respectively, frequency of electromagnetic radiation) can only be harnessed, for inference or estimation, when we know exactly how measurements are generated. Clearly, this also means that estimates of conduction delays have to be qualified; because they are conditioned on the particular generative model (i.e., DCM) in play.

The four populations are coupled with intrinsic connections that correspond (roughly) to known inter-and intralaminar connectivity (Thomson and Bannister, 2003). The anatomical and physiological evidence for this canonical microcircuit is reviewed in (Bastos et al., 2012) from the perspective of predictive coding architectures in the brain. A subsequent simplification for dynamic causal modelling of local field potentials is described in (Bastos et al., 2015). The microcircuitry in Fig. 1 includes additional (interlaminar) connections, from the superficial to the deep pyramidal cells, which are known to be prevalent in most cortical areas (Thomson and Bannister, 2003, Binzegger et al., 2004, Haeusler and Maass, 2007, Heinzle et al., 2007).

In brief, experimental and extrinsic input arrives at granular layer 4, targeting spiny stellate cells. These populations then project to superficial pyramidal cells, which project to deep pyramidal cells. In addition to this intrinsic feedforward pathway (Thomson and Bannister, 2003), there are reciprocal connections with inhibitory interneurons, modelled here with a single population. Extrinsic efferents come in two flavours. Forward connections arise from superficial pyramidal cells, while backward connections arise from deep pyramidal cells (Felleman and Van Essen, 1991, Hilgetag et al., 2000). Extrinsic forward connections provide the input to the granular layer of a higher region (with a small projection to deep pyramidal cells), while extrinsic backward connections target superficial pyramidal cells and inhibitory interneurons. Note that the recurrent (self) connections are universally inhibitory; irrespective of whether the neuronal population is excitatory or inhibitory. We assume that recurrent or self-connections in excitatory neural populations (populations 1, 2 and 4) are mediated by inhibitory interneurons; e.g., fast spiking parvalbumin positive cells (Sohal et al., 2009, Kann et al., 2014), and that the strength of the recurrent connection is determined by the level of excitation of the neuronal population itself. These connections are denoted by the pink circles in Fig. 1.

### Neurovascular coupling

Pre-synaptic activity at each subpopulation is assumed to drive haemodynamic responses, through local collaterals innervating astrocytes, whose (endfeet) processes release vasodilatory signals (Carmignoto and Gomez-Gonzalo, 2010, Figley and Stroman, 2011). These signals then enter a standard haemodynamic model to generate a BOLD signal. The hemodynamic model has been described extensively in previous communications (Friston et al., 2000) and completes the Balloon model (Buxton et al., 2004). In brief, a neurovascular signal (e.g., intracellular calcium in astrocytes) drives a vasodilatory signal (e.g., nitric oxide) that is subject to auto-regulatory feedback (Friston, 1995, Attwell and Iadecola, 2002). Blood flow responds in proportion to the vasodilatory signal and causes change in blood volume and deoxyhemoglobin content. The observed BOLD signal is a nonlinear function of volume and deoxyhemoglobin and depends upon the relative contribution of intra-and extravascular components (Buxton et al., 2004). In this model, outflow is a function of volume $F(h_{3})=h_{3}^{1/\alpha }$ and Grubb's exponent α. The relative oxygen extraction $E(h_{2})=\left(1-{\left(1-\varphi \right)}^{1/h_{2}}\right)/\varphi$ is a function of flow, where ϕ is resting oxygen extraction fraction. A description of the parameters of this model is provided in Table 2, Table 3.

**Table 2:** Haemodynamic parameters.

	**Description**	**Parameterisation**	**Prior**
η	Rate of vasodilatory signal decay per sec	$0.64\cdot \exp(\theta_{\eta })$	$p(\theta_{\eta })=N(0,\frac{1}{256})$
χ	Rate of flow-dependent elimination	$0.32\cdot \exp(\theta_{\chi })$	$p(\theta_{\chi })=N(0,0)$
τ	Rate hemodynamic transit per sec	$2.00\cdot \exp(\theta_{\tau })$	$p(\theta_{\tau })=N(0,\frac{1}{256})$
α	Grubb's exponent	$0.32\cdot \exp(\theta_{\alpha })$	$p(\theta_{\alpha })=N(0,0)$
ε	Intravascular: extravascular ratio	$1.00\cdot \exp(\theta_{\varepsilon })$	$p(\theta_{\varepsilon })=N(0,\frac{1}{256})$
φ	Resting oxygen extraction fraction	$0.40\cdot \exp(\theta_{\varphi })$	$p(\theta_{\varphi })=N(0,0)$
$\beta_{ij}$	Sensitivity of signal to neural activity	$\theta_{i}$	$p(\theta_{i})=N(0,\frac{1}{16})$

**Table 3:** Biophysical parameters.

	**Description**	**Value**
$V_{0}$	Blood volume fraction	0.08
$k_{1}$	Intravascular coefficient	6.9⋅φ
$k_{2}$	Concentration coefficient	ε⋅φ
$k_{3}$	Extravascular coefficient	1−ε

From our perspective, the important part of this model is the neurovascular coupling; namely, how neuronal activity induces a neurovascular signal; e.g., calcium transients in astrocytes (Bazargani and Attwell, 2016). We have parameterised this coupling under the assumption that there is a laminar-specific drive to the neurovascular signal mediated by collaterals of both intrinsic and extrinsic connectivity. In other words, we assume that every presynaptic input to a neuronal population is accompanied by a collateral input of the same strength to nearby astrocytes (Figley and Stroman, 2011). This enables us to parameterise laminar-specific contributions to neurovascular signals, while preserving the distinction between extrinsic and intrinsic input. Note that intracellular calcium can be influenced by both excitatory and inhibitory input to astrocytes via their glutamatergic and GABAergic receptors (Bazargani and Attwell, 2016). We therefore distinguish between excitatory and inhibitory input by assigning separate neurovascular coupling parameters to each sort of presynaptic drive (see Fig. 1).

Clearly, there could be other neurovascular architectures; for example, each population could send signals to the local vasculature in proportion to its postsynaptic activity. Alternative mechanisms such as extra-synaptic communication could also be considered. Although we do not pursue this here, it would be an interesting exercise to adjudicate among different forms of neurovascular coupling (Carmignoto and Gomez-Gonzalo, 2010, Figley and Stroman, 2011, Bazargani and Attwell, 2016) using Bayesian model comparison. In this paper, we use an over parameterised model of presynaptic neurovascular coupling for three reasons: first, it allows us to ask whether intrinsic, extrinsic or both sorts of presynaptic afferents are responsible for eliciting a neurovascular response; this illustrates the latitude afforded by having an explicit model of neurovascular coupling. Second, it allows us to distinguish between the contribution of excitatory and inhibitory collaterals. Third, having a laminar specific neurovascular parameterisation may be useful in the future when modelling high resolution (laminar) fMRI (Heinzle et al., 2016). In future applications of canonical microcircuit DCM to fMRI data, we anticipate that this part of the model will become much simpler and informed; particularly when using Bayesian fusion of EEG and fMRI data to resolve conditional dependencies between neuronal and haemodynamic parameters. This completes our specifications of the model for a single node. We now consider how nodes are assembled to form a graph with directed forward and backwards connections.

### Dynamic causal models for graphs

**Fig. 2:**
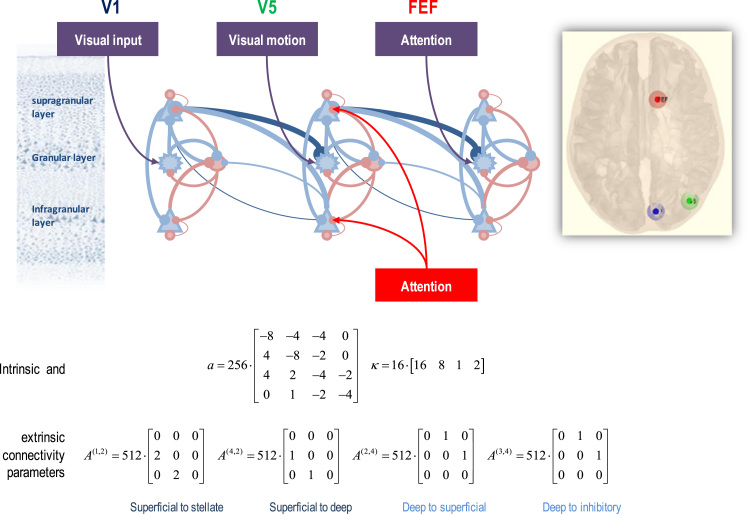
This figure provide an example of the extrinsic connectivity architecture used in this sort of DCM – and the particular network or graph used in this paper. Here, we have selected three regions that comprise an early visual source (V1), a motion sensitive area (V5 or MST) and an attentional area; the frontal eye fields (FEF). Forward connections arise primarily from superficial pyramidal cells and target spiny stellate cells in the granular layers. In addition, we have modelled a (lower density) connectivity to deep pyramidal cells. Backward connections arise from deep pyramidal cells and target inhibitory interneurons and superficial pyramidal cells. The laminar specificity of these extrinsic connections is specified quantitatively by the prior expectations of the connectivity parameters in the lower equalities. In addition to specifying the extrinsic connectivity architecture, it is necessary to specify where experimental inputs drive or modulate neuronal responses. Here, *visual input*, *visual motion* and *attention* drive responses in the early visual cortex, motion sensitive cortex and frontal eye fields respectively. Crucially, attention exerts a modulatory effect on the self-inhibition of superficial and deep pyramidal cells in the hierarchically intermediate area (V5). Our key question was whether the attentional modulation of superficial, deep or both pyramidal populations is necessary to explain the observed data. Please see the tables for a description of the variables in this figure.

A distributed network or graph is determined by between-node or extrinsic connectivity. The distinction between forward and backward connections defines the hierarchical relationships among cortical and subcortical systems – and has clear correlates in terms of laminar-specific origins and targets (please see above). This hierarchical aspect of the current DCM was missing from earlier variants based upon Taylor approximations. This is because earlier versions did not model laminar-specific neuronal dynamics. Fig. 2 illustrates the extrinsic connectivity architecture, in terms of the network that will be used to analyse empirical data in the next section.

Here, we have selected three areas that showed a significant experimental effect (either attentional modulation or motion sensitive responses – see below) shown in the inset. These comprise an early visual source (V1) a motion sensitive area (V5 or MST) and an attentional area; the frontal eye fields (FEF). These three nodes are modelled as a simple hierarchy, where forward connections arise from superficial pyramidal cells and target spiny stellate cells in the granular layers of a higher level. In addition, we have modelled a (weak) connectivity to deep pyramidal cells. Backward connections arise from deep pyramidal cells and target inhibitory interneurons and superficial pyramidal cells in the level below. The laminar specificity of these extrinsic connections is specified quantitatively by the prior values of the parameters of adjacency or **A** matrices shown in the lower panel of Fig. 2. See also Table 1. These extrinsic adjacency matrices model two sorts of forward connections (to spiny stellate and deep pyramidal cells and the two sorts of backward connections (to superficial pyramidal cells and inhibitory interneurons) respectively. While this represents only a subset of known connections within the visual hierarchy; e.g. Ninomiya et al. (2012), our aim here was to specify a plausible and minimal subgraph that would support questions about attentional modulation within V5. Hypotheses about different extrinsic connectivity architectures can be tested in the usual way using Bayesian model comparison with different **A** matrices.

In addition to specifying the extrinsic connectivity architecture, it is necessary to specify where experimental inputs (here, *visual input*, *visual motion* and *attention* to visual motion) drive or modulate neuronal responses. As usual, the driving effects of experimental inputs are determined by the parameters of a **C** matrix, directing experimental input to spiny stellate populations in each region. In this example, visual input, visual motion and attention drive responses in the early visual cortex, motion sensitive cortex and frontal eye fields respectively. Finally, we have to specify where experimental effects change intrinsic or extrinsic connectivity. For each experimental effect there are a pair of **B** matrices whose off-diagonal terms parameterise the change in the (forward or backward) adjacency matrix per-unit change in experimental input. The diagonal elements of the forward **B** matrix are used to model experimental modulation of intrinsic (recurrent or self-inhibition) of superficial pyramidal cells, while the diagonal elements of the backward **B** matrix encode condition specific changes in the self-inhibition of deep pyramidal cells. In this example, attention exerts a modulatory effect on both the self-inhibition of superficial and deep pyramidal cells in area (V5). This particular parameterisation allowed us to ask whether attentional modulation of superficial, deep or both pyramidal cell populations is necessary to explain the observed responses.

On a general note, the **B** matrices usually contain the parameters of greatest interest in DCM. This is because they mediate (experimentally induced) context sensitive changes in coupling. In other words, they model the *dynamic effective connectivity*, which characterises functional integration in the brain (Jirsa et al., 2010, Senden et al., 2014). Clearly, the parameterisation in DCM is relatively simple. This is because experimental inputs can change the strength of extrinsic or intrinsic connections (i.e., change the excitability of neuronal populations in receipt of specific afferents). In reality, these effects would themselves be mediated by other brain systems that, in principle, could be included in the model. This is the ambition of nonlinear generalisations of vanilla (bilinear) DCM for fMRI. See Stephan et al. (2008) for a fuller discussion.

## An empirical illustration: attentional modulation of intrinsic connectivity

In this section, we use the exemplar DCM in Fig. 2 to analyse empirical data from a single subject. These data were acquired over 20 years ago – and have been used for demonstrations and training purposes since that time (Buchel and Friston, 1997). Therefore, the analyses presented below are for didactic purposes to show how questions can be framed and answered. In what follows, we briefly describe the data and the results of dynamic causal modelling.

### Data and whole brain analysis

Timeseries data were acquired from a normal subject at 2 T using a Magnetom VISION (Siemens, Erlangen) whole body MRI system. Contiguous multi-slice images were acquired with a gradient echo-planar sequence (TE=40 ms; TR=3.22 seconds; matrix size=64x64×32, voxel size 3x3×3 mm). Four consecutive hundred-scan sessions were acquired, comprising a sequence of 10-scan blocks under five conditions. The first was a dummy condition to allow for magnetic saturation effects. In the second, *Fixation*, the subject viewed a fixation point at the centre of the screen. In an *Attention* condition, the subject viewed 250 dots moving radially from the centre at 4.7° per second and was asked to detect changes in radial velocity. In *No attention,* the subject was asked simply to view the moving dots. In last condition, the subject viewed stationary dots. The order of the conditions alternated between *Fixation* and photic stimulation. In all conditions the subject fixated the centre of the screen. No overt response was required in any condition and there were no actual speed changes.

The three potential causes of neuronal activity were encoded as box-car functions corresponding to the presence of a visual stimulus, motion in the visual field and attention. A standard whole brain SPM analysis then identified several regions showing a significant effect of visual input, motion and attention (see the inset in Fig. 2). From these, we selected a region showing an effect of visual stimulation (early visual cortex, labelled V1); a region showing the effect of visual motion (V5) and a region showing an effect of attention (FEF). Regional activity was summarised with the principle eigenvariate of all significant (p<0.001 uncorrected) voxels within 8 mm of the most significant voxel within each region. These timeseries were subject to dynamic causal modelling in the usual way but using the canonical microcircuit model of the previous section.

The priors on the parameters of the neural mass model are provided in Table 1. Some of these parameters have zero prior variance; in other words, in contrast to DCM for electromagnetic data, we fix some parameters to their prior mean, because they cannot be estimated efficiently in the context of MRI. Clearly, if one had also performed the experiment using EEG, these priors could be replaced by the posterior means and covariances following a standard DCM analysis of evoked or induced electromagnetic responses. This corresponds to the Bayesian fusion or belief updating mentioned in the introduction.

In these sorts of DCM, most of the unknown variables are scale parameters. In other words, they never take negative values (for example, connection strengths and synaptic rate constants). This means the free parameters of a DCM are generally log-scaling parameters, where a value of zero means a scaling of exp(0)=100%. The variance of a Gaussian shrinkage prior therefore determines to what extent the scaling of a particular value can deviate from its prior log-scaling of zero. The prior variances in Table 1 ensure that free parameters are restricted in their scaling to within an order of magnitude or less.

### Dynamic causal modelling

**Fig. 3:**
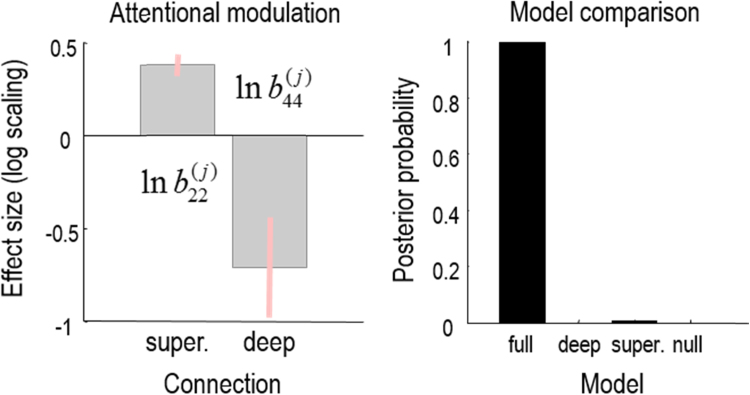
This figure shows the results of inference about attentional modulation. The left panel shows the posterior density over the two modulatory effects on superficial and deep pyramidal cells in motion sensitive area V5. The posterior mean is the grey bar, while the 90% posterior confidence intervals are shown in pink. One can see that both (log scaling) effects are substantially greater and smaller than the prior mean of zero (i.e. a scaling of 100%). The intuition that both parameters are necessary to explain the observed responses is confirmed through Bayesian model comparison. The right panel shows the results of Bayesian model reduction of the full model, when eliminating either the modulation of the superficial pyramidal cells, deep pyramidal cells or both. With these data, we can be almost 100% confident that both effects are evident in these data.

Fig. 3 shows the results of inference about the effects of attention. The left panel shows the posterior density over the two modulatory effects on superficial and deep pyramidal cells in motion sensitive area V5. The posterior expectations are shown as grey bars, while the 90% posterior confidence intervals are shown in pink. One can see immediately that both (log-scaling) effects are substantially greater and smaller than their prior mean of zero. The intuition that both parameters are necessary to explain the observed responses is confirmed through Bayesian model comparison: the right panel shows the results of Bayesian model reduction of the full model when eliminating either the modulation of superficial pyramidal cells, deep pyramidal cells or both. With these data, we can be almost 100% confident that both effects are evident.

Neurobiologically, this is interesting because there are neuromodulatory synaptic processes that implicate both superficial and deep pyramidal cells. For example, cholinergic modulation of inhibitory interneurons is usually associated with the modulation of superficial (and deep) pyramidal cells through their interactions with inhibitory interneurons (Everitt and Robbins, 1997, Kopell et al., 2011, Lee et al., 2013, Vossel et al., 2014, Hedrick and Waters, 2015). Conversely, the laminar specific deployment of nicotinic (cholinergic) receptors and 5HT receptors generally implicate deep pyramidal cells (Hedrick and Waters, 2015, Nocjar et al., 2015). See Auksztulewicz and Friston, 2015, Vossel et al., 2015 for a further discussion of the importance of laminar specific attentional modulation in the context of predictive coding and precision control. These studies used the canonical microcircuit neural mass model above to analyse EEG and MEG data. Having briefly illustrated the sort of application we envisage people might want to pursue, we now turn to the haemodynamic parameters that can only be estimated using fMRI.

## Bayesian model comparison and neurovascular coupling

**Fig. 4:**
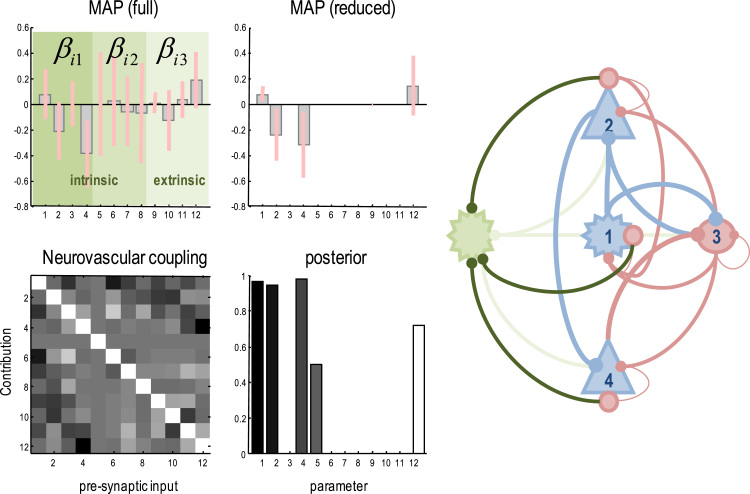
This figure shows the characterisation of neurovascular coupling in terms of the parameters that couple afferent presynaptic activity to the neurovascular signal in each region. The inset (on the right) shows that inhibitory presynaptic collaterals from the excitatory neuronal populations are the most important. Estimates of the neurovascular coupling parameter are shown on the upper left using the same format as the previous figure. These 12 parameters correspond to intrinsic inhibitory collaterals (shown on dark green) intrinsic excitatory collaterals (shown on green) and extrinsic excitatory collaterals (shown on light green) to each of the four populations. These collaterals provide distinct inputs because the posterior correlations among the associated parameter estimates are not intense (see lower left panel). The Bayesian parameter averages – following Bayesian model reduction over all combinations of the 12 neurovascular coupling parameters – are shown on the upper right. This procedure shrinks redundant parameters to their prior expectation (of zero). The lower right panel shows the posterior probability over all models with and without each of the 12 parameters. These Bayesian parameter averages suggest that, in this instance, intrinsic inhibitory activity is the most important determinant of haemodynamic responses.

Fig. 4 characterises neurovascular coupling in terms of the parameters that couple presynaptic activity to the neurovascular signal in each region. The inset (on the right) summarises the results of Bayesian model averaging over all reduced models; which considered all combinations of the 12 neurovascular coupling parameters. The Bayesian model averages suggest intrinsic inhibitory collaterals (to excitatory populations) were the most potent in eliciting a neurovascular signal. In more detail, the parameter estimates of the full model – with all 12 parameters – are shown on the upper left using the format of Fig. 3. The 12 neurovascular coupling parameters correspond to intrinsic inhibitory collaterals (dark green) intrinsic excitatory collaterals (green) and extrinsic excitatory collaterals (light green) to each of the four populations. Interestingly, each set of inputs appears to provide complementary information, because the conditional or posterior correlations among the parameters are mild (see lower left panel). The Bayesian parameter averages (Hoeting et al., 1999) – following Bayesian model reduction over all combinations of the 12 parameters – are shown on the upper right. This procedure shrinks redundant parameters to their prior expectation (of zero) (Friston et al., 2016).

The lower right panel shows the posterior probability over all models with and without each of the 12 parameters. These Bayesian parameter averages suggest that, in this instance, intrinsic inhibitory collaterals are the most important determinant of haemodynamic responses. Interestingly, these parameter estimates are both positive and negative. This suggests that (self-inhibiting) collaterals from the excitatory populations may target astrocytes to both increase and decrease local blood flow. Irrespective of the precise arrangements of axonal collaterals (or heterosynaptic facilitation), this sort of result highlights the key role of inhibitory interneurons not only in mediating synchronisation and excitability (Kopell et al., 2011) – but also in orchestrating neurovascular responses (Kann et al., 2014, Anenberg et al., 2015).

Having said this, it is important to remember that this result should not be generalised. If one was really interested in the form of neurovascular coupling, one would use a much more informed model of laminar specific neuronal responses based upon extensive DCM studies using EEG. For example, one could use a DCM for cross spectral responses (Moran et al., 2008, Friston et al., 2012) in each of the conditions studied with fMRI to provide informative (empirical) priors for the canonical microcircuit. This Bayesian fusion or updating should then provide much more efficient estimates of the haemodynamic parameters – and a more definitive Bayesian model comparison. We anticipate that this sort of analysis will be used to refine the model of neurovascular coupling – that could then be used in routine applications of canonical microcircuit DCMs.

## The electrophysiological correlates of haemodynamic responses

**Fig. 5:**
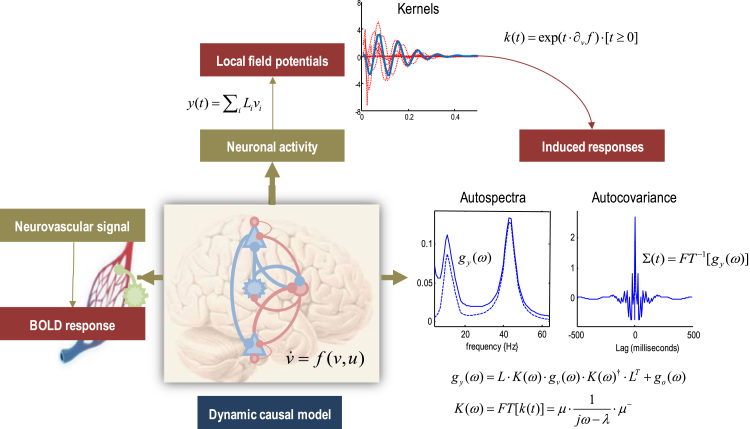
This schematic illustrates the potential for generating multimodal predictions from the same (neuronal) dynamic causal model. The previous figures focused on the generation of BOLD responses that are mediated by (hidden) neuronal states. However, these states can also be used to generate predictions of the local field potentials or event related responses; here, characterised through a linear mapping with a standard electromagnetic lead field. The first-order kernel mapping from experimental input to predicted electrophysiological responses (at the top of the figure) is what would be seen in response to a very brief stimulus. Under local linearity assumptions, one can use these kernels to predict induced responses that are generated by random fluctuations about the mean neuronal activity. This means that, given the spectral density of neuronal fluctuations, one can generate induced responses. These are illustrated on the right of the figure in terms of the autospectra (with and without observation noise in solid and dashed lines respectively) and the associated autocovariance function (i.e., the Fourier transform of the autospectra). The equations in this figure show the relationships between the first-order kernels, cross spectral density and covariance functions used to generate these sorts of predictions. Please see Table 4 for a full description of the expressions.

In this section, we use the parameter estimates of the neuronal model to generate local field potential responses, to characterise the electrophysiological correlates of the observed BOLD responses. Fig. 5 provides a schematic that illustrates the generation of multimodal predictions from the same dynamic causal model. The previous sections focused on the generation of BOLD responses that are mediated by (hidden) neuronal states. However, these states can also be used to generate predictions of local field potentials or event related responses; here, characterised as a linear mixture of superficial and deep pyramidal cell depolarisation (here, superficial pyramidal cells and deep pyramidal cells contributed in equal amounts).

The resulting first-order kernel – mapping from experimental input to predicted electrophysiological responses – is what one would expect to see in response to a very brief stimulus. Under local linearity assumptions, one can use these kernels to predict induced responses that are generated by random fluctuations about the mean evoked response (modelled by the first-order kernel). This means that, given the spectral density of neuronal fluctuations, one can generate induced responses. These are illustrated on the right, in terms of autospectra (with and without observation noise in solid and dashed lines respectively) and the associated autocovariance function (i.e., the Fourier transform of the autospectra). The equations in Fig. 5 show the relationships between the first-order kernels, cross spectral density and covariance functions used to generate these sorts of predictions. In short, these relationships enable one to generate evoked and induced (spectral) electrophysiological responses starting with the neuronal parameters of a canonical microcircuit; even when the parameters have been optimised using fMRI. Please see Table 4 for a glossary of expressions used in this figure and (Moran et al., 2008, Friston et al., 2012, Friston et al., 2014a) for a fuller description of the underlying linear systems theory.

**Table 4:** Glossary of variables and expressions.

**Variable**	**Description**
$u_{m}$	The *m*-th of *M* experimental inputs as a function of time
$v_{i}^{\left(j\right)}$	The *i*-th (neuronal) state in region *j*; e.g., mean depolarisation of a neuronal population
$\sigma (v_{i}^{\left(j\right)})$	The neuronal firing rate – a sigmoid squashing function of depolarisation
$q_{i}^{\left(j\right)},p_{i}^{\left(j\right)},r_{i}^{\left(j\right)}$	(intrinsic, extrinsic and combined) presynaptic input to the *i*-th population of region *j*; eliciting postsynaptic and neurovascular responses; e.g., by depolarising astrocytes
s	Neurovascular signal; e.g., intracellular astrocyte calcium levels
$h_{1},h_{2},h_{3},h_{4}$	Haemodynamic states: h_1_ - vasodilatory signal (e.g., NO), h_2_ - blood flow, h_3_ - blood h_4_ - volume deoxyhaemoglobin content
$L_{i}$	Lead field vector mapping from (neuronal) states to measured (electrophysiological) responses
$g_{v}(\omega ),g_{o}(\omega ),g_{y}(\omega )$	Spectral density of (neuronal) state fluctuations, observation error and ensuing measurement, respectively
$\partial_{x}f$	System Jacobian or derivative of system flow with respect to (neuronal) states
k(t)=FT[K(ω)]	First order kernel mapping from inputs to responses; c.f., an impulse response function of time. This is the Fourier transform the transfer function
K(ω)=FT[k(t)]	Transfer function of frequency modulating the power of endogenous neuronal fluctuations to produce a (cross spectral density) response. This is the Fourier transform of the kernel
λ	Eigenvalues of the transfer function
$\mu ,\mu^{-}$	Eigenvectors of transfer function and their generalised inverse

**Fig. 6:**
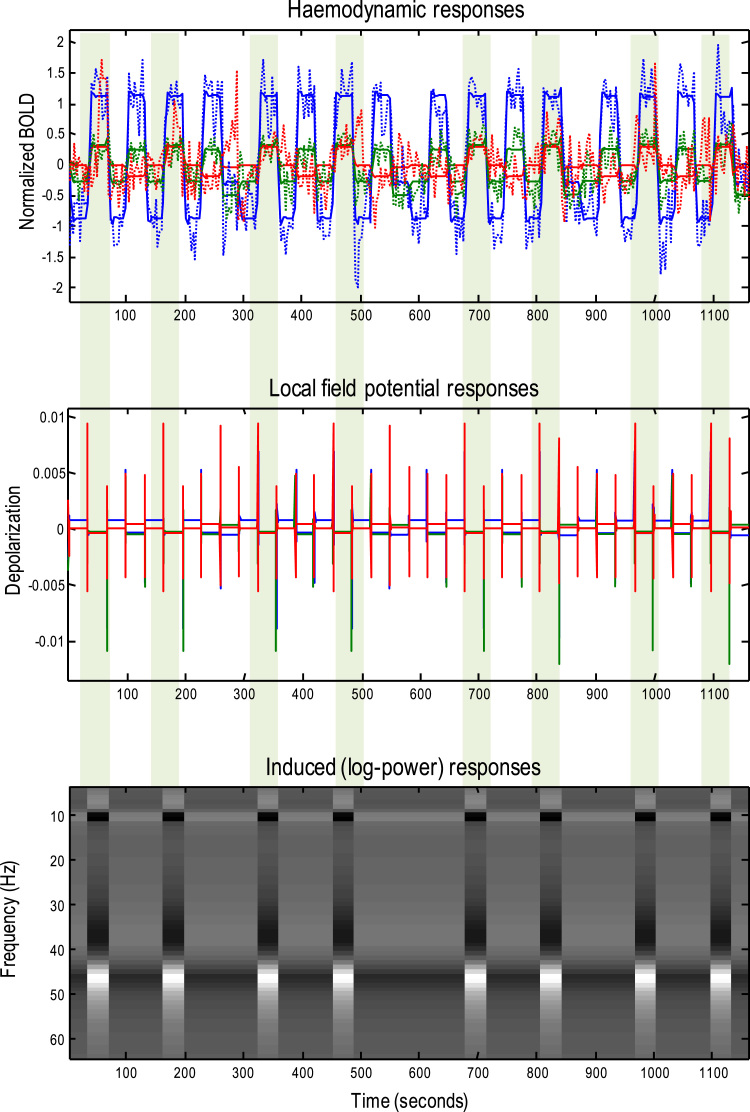
This figure shows the predicted and observed BOLD responses in each of the regions (upper panel) accompanied by simulated (but unobserved) local field potentials (middle panel). In addition, the time frequency induced responses are shown for the motion sensitive region (V5), over the entire session. The agreement between the predicted (solid lines) and observed (dotted lines) fMRI responses is self-evident. The blue lines correspond to the early visual response (V1) which shows little attentional modulation. Conversely, the motion sensitive area (V5, green lines) shows a profound motion sensitive response that is modulated by attention by about 10%. The frontal eye field responses (red lines) show a marked attentional modulation but little in the way of visual selective responses. The electrophysiological responses show a similar profile; illustrating large offset and onset responses and then maintenance at the fixed point for each level of experimental input. The attentional modulation of the superficial and deep pyramidal cells in the motion sensitive area changes the connectivity and subsequent predictions of induced responses. These are entirely consistent with alpha (at 10 Hz) desynchronization during attention that is accompanied by an increase in gamma activity (at 48 Hz). The genesis of these induced responses is addressed in more detail in the next figure. The light green bars indicate periods of attention to visual motion.

The generation of predicted spectral or induced responses inherits the procedures used in DCM for cross spectral density (Friston et al., 2012). In brief, the endogenous neuronal fluctuations are assumed to have a scale-free form that is parameterised by (log) exponents. These assumptions allow us to simulate induced responses during different experimental conditions or blocks in the fMRI experiment. In other words, not only can we reproduce the fMRI signal used to estimate these parameters but one can also generate the electrophysiological signals that would have been seen if these parameters were correct. Fig. 6 shows the predicted and observed BOLD responses in each of the regions (upper panel) accompanied by simulated (but unobserved) local field potentials (middle panel). In addition, time frequency induced responses are shown for the motion sensitive region (V5) over the entire session. The agreement between the predicted (solid lines) and observed (dotted lines) fMRI responses is self-evident. The blue lines correspond to the early visual response that, although vigorous, shows little attentional modulation (with a slight deactivation during static visual stimulation). Conversely, the motion sensitive area (green) shows a profound motion sensitive response that is modulated by attention by about 10%. The frontal eye field responses show a marked attentional modulation but little in the way of visual selective responses.

The electrophysiological responses show a similar profile; illustrating large offset and onset responses and then maintenance at their fixed point for each condition-specific profile of experimental input. The attentional modulation of the superficial and deep pyramidal cells in the motion sensitive area changes the connectivity and subsequent predictions of induced responses. These are consistent with alpha (at 10 Hz) desynchronization during attention that is accompanied by a marked increase in gamma activity (at 48 Hz) (Fries, 2005, Bauer et al., 2006, Lee et al., 2013, Bauer et al., 2014, Buschman and Kastner, 2015).

**Fig. 7:**
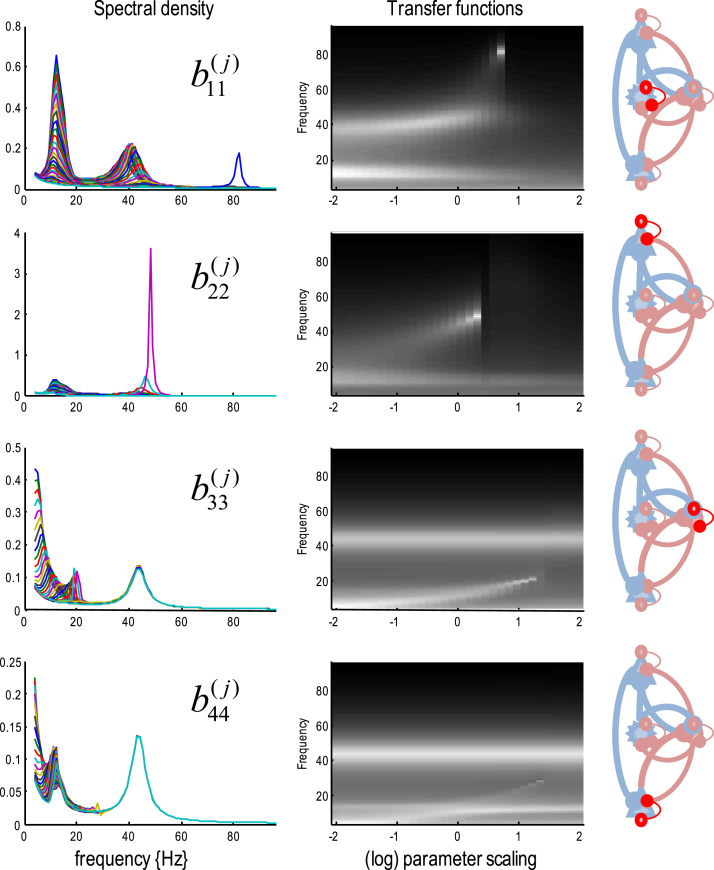
This figure shows the effect of modulating the self-inhibition of each of the four subpopulations (in the absence of afferent or experimental input). Each row shows the autospectra from each of the four populations (spiny stellate, superficial pyramidal, inhibitory interneurons and deep pyramidal cells respectively) over a log scaling from −2 to +2. The left panels show the resulting autospectra from 0 to 96 Hz, while the right panels show the same data in image format. These suggest that increasing the self-inhibition of spiny stellate cells rapidly suppresses alpha activity and increases the frequency of gamma activity until a bifurcation at a peak gamma activity of about 80 Hz. This phase transition is seen even earlier as the self-inhibition of superficial pyramidal cells increases, with a peak gamma of about 42 Hz. The effects of increasing self-inhibition of inhibitory interneurons and deep pyramidal cells are to suppress alpha activity and convert it into fast activity. See main text for further discussion.

The genesis of these induced responses is addressed in more detail in Fig. 7. This figure shows the effect of modulating the self-inhibition of each of the four subpopulations (in the absence of afferent or exogenous input). Each row shows the autospectra from the four populations (spiny stellate, superficial pyramidal, inhibitory interneurons and deep pyramidal cells respectively) over a log scaling from −2 to +2. In other words, from 0.13 to 7.38 times the prior mean. The left panels show the resulting autospectra, while the right panels show the same data in image format. These results show that increasing the self-inhibition of spiny stellate cells rapidly suppresses alpha activity and increases the frequency of gamma activity until a (transcritical) bifurcation at a peak gamma activity of about 80 Hz. This phase transition is seen even earlier as the self-inhibition of superficial pyramidal cells increases, with a peak gamma of about 42 Hz. The effects of increasing self-inhibition of inhibitory interneurons and deep pyramidal cells are to suppress alpha activity and convert it into fast activity: c.f., (Lee et al., 2013, Kann et al., 2014). With these characteristics in mind, one can now see why increasing the gain (self-inhibition) of superficial pyramidal cells – in conjunction with a decrease in self-inhibition of deep pyramidal cells – attenuates alpha activity, while increasing the amplitude and frequency of gamma activity. Although a simplistic interpretation of increasing self-inhibition (e.g., of superficial pyramidal cells) could be construed as reducing its excitability, the emergent responses in the setting of interactions with inhibitory interneurons and other populations within the canonical microcircuit produce a desynchronization that is more reminiscent of an activation (Pfurtscheller et al., 1996, Lee et al., 2013).

## Discussion

This paper has described a denouement of dynamic causal modelling for fMRI timeseries; in the sense that it combines neurophysiologically plausible models of neuronal processing and established haemodynamic models of fMRI signals. To briefly rehearse the advantages of this modelling, we note that the sorts of questions addressed by DCM for electromagnetic neurophysiology can now, in principle, be addressed to fMRI data.

Perhaps the most important advantage has yet to be exploited; namely, the opportunity to use EEG or MEG data in conjunction with fMRI data to doubly inform parameter estimation and model selection at the level of neuronal architectures. A straightforward strategy suggests itself along the following lines: one first identifies the key regions engaged by an experimental paradigm using standard whole brain (SPM) analysis of fMRI data. Significantly activated regions can then be modelled as a neuronal network to explain evoked or induced electromagnetic responses (or both), using the location of activated regions as prior locations in DCM for ERP or cross spectral density responses. This modelling then provides precise or informative posterior densities over neuronal (effective connectivity and synaptic) parameters, which are passed as priors to a subsequent DCM of the fMRI data. The fMRI data supply informative constraints on the haemodynamic parameters; thereby providing a comprehensive characterisation of how non-invasive brain signals are generated. Note that this Bayesian data fusion does not rely upon concurrent EEG and fMRI. This enables the optimisation of both protocols that are linked through a common experimental manipulation. We hope to illustrate this approach elsewhere, using another (training) dataset based upon the viewing of familiar faces (Wakeman and Henson, 2015); for which we have EEG, MEG and fMRI data. In what follows, we consider some practical and strategic issues.

### Practical issues

Readers familiar with extant DCM for fMRI procedures will note that there are now four adjacency or **A** matrices, as opposed to a single **A** matrix in standard DCM. This follows from the fact that there is a distinction between forward and backward connections – and that within forward and backward connections there are two collateral streams. In practice, the user specifies allowable connections in two adjacency matrices, specifying whether a forward and backward connection exists. These prior specifications are then applied to both sorts of forward and backward connections respectively. Note that it is possible to have both forward and backward connections between a target and source region. These are often used to model lateral connections that have a less specific (usually bilaminar) laminar specificity (Markov et al., 2013). Similarly, one has to specify pairs of **B** matrices for each experimental effect. Crucially, enabling condition specific effects in the diagonal elements of these (forward and backward) matrices is interpreted as allowing the modulation of the self inhibition of (superficial and deep) pyramidal cells respectively. Other effects are easy to implement by simply changing the equations of motion (in **spm_gen_fmri.m**).

For readers who require a more technical (annotated) description of the procedures, a demo routine (**DEMO_dcm_fmri_nnm.m**) is available in the current version of the SPM software that reproduces the graphics in this paper. This routine shows how the core inversion routine (**spm_dcm_fmri_nmm.m**) is called and how to specify the requisite input arguments. In terms of numerics, the inversion of a typical timeseries takes about 16 s per iteration on a standard personal computer, where 32 iterations are usually required for convergence. One might ask how one can solve or integrate the neuronal equations of motion so quickly, over the long periods of time typically associated with an fMRI session. The computational efficiency follows from the fact that the neural mass model has a fixed point that is reached within a second or so, following any change in experimental or exogenous input. This means that the neuronal equations of motion only have to be solved for a brief period of time, following any change in experimental condition. Crucially, these changes are relatively infrequent in standard fMRI paradigms, from the point of view of neuronal dynamics that unfold over time scale of several hundred milliseconds. In block designs (of the sort used in this paper), one is effectively using an adiabatic approximation to neurodynamics. In other words, for the majority of the time, neuronal activity has attained steady-state, because neuronal dynamics are very fast (on a scale of milliseconds) in relation to changes in input (every few seconds). As an alternative, fast GPU based integration of the differential equations could be adopted for paradigms with very frequent input changes (Aponte et al., 2016). Note that the transients in the middle panel of Fig. 6 are short-lived, in relation to the periods of steady-state activity attained during each block. This means the neuronal transients contribute relatively little to sustained BOLD responses.

Practically, this means a canonical microcircuit DCM for fMRI can be applied to relatively small graphs (with eight nodes or less) in a reasonable amount of time. However, the current inversion scheme may take many hours with larger graphs – and may call for additional constraints that finesse the computational load; e.g., Seghier and Friston (2013) or an equivalent canonical microcircuit formulation for cross spectral data features; e.g., Razi et al. (2015). The practical issue here is the computation of (variational free energy) gradients with respect to parameters that increases the quadratically with a number of nodes. Theoretically, there is no upper bound on the number of parameters that can be estimated; indeed, increasing the number of parameters usually eludes local minima problems. Furthermore, redundant parameters can be removed easily, using Bayesian model reduction (Friston et al., 2016). Having said this, the computational burden of inverting a full model remains an issue for large DCMs.

### Strategic issues

Perhaps the most interesting insight (at least for the authors) offered by the modelling in this paper pertains to the electrophysiological correlates of BOLD activity (and *vice versa*). We had always imagined that there was some systematic (unique or diffeomorphic) mapping between induced electrophysiological responses and concurrent fMRI signals (Kilner et al., 2005). In other words, we had always assumed that there was some generalised convolution of induced (time frequency) electrophysiological signals that would predict BOLD activity (Logothetis et al., 2001, Laufs et al., 2003). The argument here goes as follows. If neuronal activity causes both induced responses and BOLD responses; then a deconvolution of induced (or BOLD) responses should reproduce the underlying neuronal activity, which can then be convolved to generate BOLD (or induced) activity. Therefore, the composition of a deconvolution and convolution should, in principle, map between induced and BOLD responses (Kilner et al., 2005). However, this argument overlooks the fact that the requisite deconvolutions are ill-posed, which means a direct mapping between induced and BOLD responses does not necessarily exist. For example, there may be many different patterns of neuronal activity that produce the same spectral responses.

**Fig. 8:**
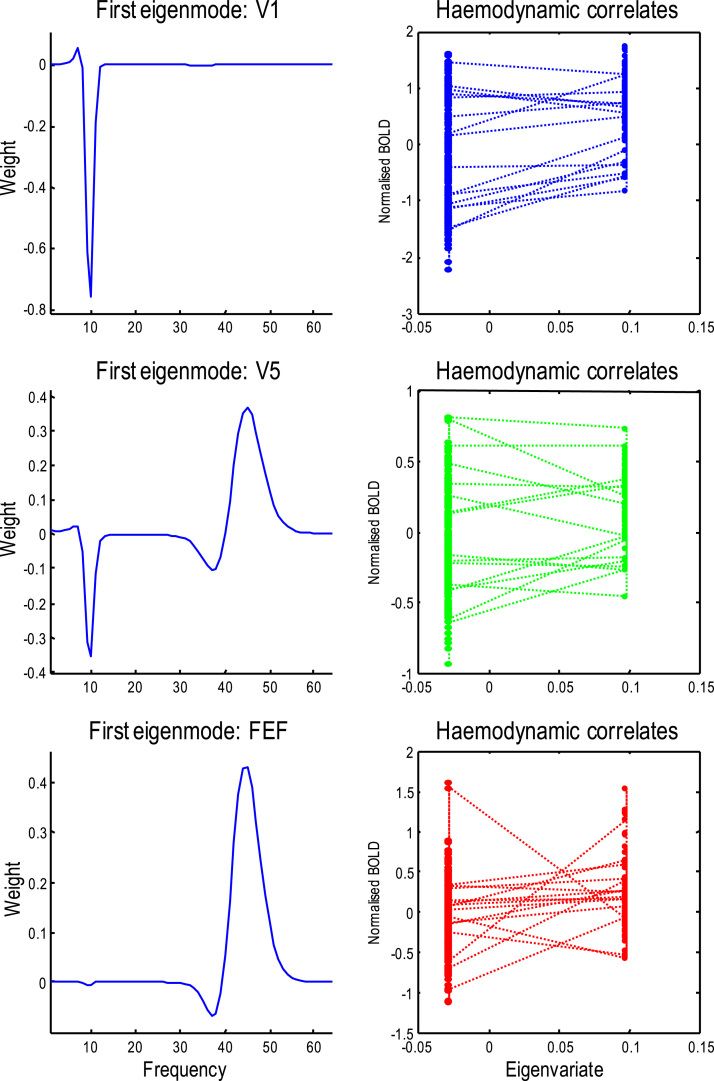
This figure highlights the (degenerate) relationship between fluctuations in spectral power and haemodynamic responses. The three rows of this figure report the responses of the three regions; namely, the early visual region, a motion sensitive region and the frontal eye fields. The left panels show the first principal component or eigenvariate of fluctuations in the power of induced responses (based upon the posterior estimates of attentional modulation in Fig. 3). The right panels plot observed haemodynamic responses against the expression of these frequency modes. The dotted lines connect consecutive time points. In the early visual cortex, there is a profound alpha suppression that is accompanied by an increase in gamma in V5. This desynchronization is limited to gamma activity in the FEF.

This insight is laid bare by the results described in the previous section: these show that there is no one-to-one (diffeomorphic) mapping between induced and BOLD responses. To make this clear, Fig. 8 highlights the complicated relationship between fluctuations in spectral power and haemodynamic responses. The three rows of this figure report the responses of the three regions modelled above. The left panel shows the pattern of frequency specific fluctuations – in responses induced by attention – in terms of the first principal component (i.e., eigenmode) of induced responses (of the sort shown in the lower panel of Fig. 6). The right panels plot observed haemodynamic responses against the expression (i.e., eigenvariate) of these frequency modes. Because the spectral responses depend (under the linearity assumptions of this DCM) on, and only on connectivity – and connectivity depends only upon the presence of attention, the induced responses have just two levels; with and without attention to motion. In the early visual cortex, there is a profound alpha suppression that is accompanied by an increase in gamma in V5. This desynchronization is limited to gamma activity in the FEF. The haemodynamic correlates (right panels in Fig. 8) are roughly consistent with the heuristic that higher bold activity is associated with the expression of higher electrophysiological responses (Kilner et al., 2005); however, these results also illustrate the fact that the BOLD activity (and underlying neuronal steady-state activity) can change markedly, without any necessary changes in spectral activity. This means that there is no necessary one-to-one relationship between spectral activity and haemodynamic responses. Clearly, more sophisticated neural mass models – with activity-dependent changes in connectivity – would finesse this problem; however, the current modelling provides a proof of principle that there is no necessary (one-to-one) relationship between the electrophysiological and haemodynamic correlates of neuronal dynamics.

A strong (radical) conclusion is that the search for the spectral (electrophysiological) correlates of haemodynamic responses is doomed to failure because electrophysiological responses do not cause haemodynamic responses and haemodynamic responses do not cause induced responses – both are caused by underlying neuronal activity. Perhaps, this should not be too surprising given the cautionary conclusion of studying endogenous brain activity as detected by surface EEG-combined fMRI: namely, "that one EEG feature can correlate with different fMRI activation maps and that a single resting state network may be associated with a variety of EEG patterns" (Laufs, 2008). All of this suggests that instead of trying to understand the relationship between EEG and BOLD measurements it is more tenable to treat both as the observable consequences of hidden neuronal activity – and try to understand how neuronal activity causes these (multimodal) measurements. This of course is the premise of the current DCM. The fact that we have a generative model of the causal relationships means we can finesse the ill-posed deconvolution above by calling on (prior) knowledge or constraints. In effect, Bayesian model inversion of a convolution model is, by definition, a (Bayesian) deconvolution.

In summary, even in the absence of a one-to-one relationship between EEG and BOLD measurements, both modalities can still inform each other, if they provide complementary constraints on model parameters. An important example here is the ability of EEG to constrain posterior estimates of canonical microcircuitry, which increases the efficiency of estimating parameters that mediate neurovascular coupling using fMRI. This increase in efficiency rests upon resolving conditional dependencies among different sets of parameters, thereby exploiting the complementary nature of both modalities. Conversely, fMRI can provide very precise constraints on parameters that are difficult to estimate in EEG and MEG. Perhaps the most obvious are the locations of functionally specialised regions or nodes that constitute the DCM. These are specified implicitly by selecting volumes of interest in DCM for fMRI; however, source locations have to be estimated in EEG – and source reconstruction is a notoriously difficult aspect of the forward problem implicit in DCM for EEG. The term *Bayesian fusion* has been introduced to emphasise the implicit handshake between different modalities – that can only be realised with a common forward model that can generate the modalities in question.

The ability to estimate microcircuitry and neurovascular parameters efficiently – and implicitly adjudicate among alternative models or hypotheses with greater precision – shifts the emphasis on the sorts of questions that one might ask. As with much of dynamic causal modelling, this shift is away from simply localising functionally differentiated responses and towards an understanding of functional integration – and how it is mediated at the synaptic level. The particular motivation for the current DCM was to answer questions about pharmacological effects that may be expressed on superficial versus deep pyramidal cells. However, one can imagine a host of interesting questions that pertain to laminar specific cortical architectures. And, more generally, the use of the fMRI to address the locus of experimental (e.g., pharmacological, attentional, pathophysiological, etc.) effects on extrinsic (long-range) changes in connectivity relative to intrinsic (short-range) coupling – and whether these are mediated primarily by inhibitory or excitatory synaptic interactions.

### Limitations

The usual limitations apply to this form of dynamic causal modelling. The most important thing to remember is that all models are wrong and there is no true model. In other words, the quality of a model – as assessed by Bayesian model comparison or reduction – depends upon the data at hand (Penny, 2012). This means there is no true model because the best model will simplify itself to match the amount of information in the data (by reducing the contribution of complexity to model evidence). The only thing that one can infer is that one model is better than another. In this sense, simply having a framework that enables model comparison at the level questions or hypotheses are posed is both sufficient and complete. To emphasise this point, any question or criticism that one can imagine – about the parameterisation of the model described in this paper – can be turned into a hypothesis and tested, using the procedures described above. For example, if one wanted to know the impact of making implausible assumptions about intrinsic connectivity, one can score the effect of different assumptions in terms of their relative model evidence. This allows one to identify the parameters and architectures that ‘make a difference’, in terms of inferences based upon the data to hand. See Troebinger et al. (2014) for an example of this application of Bayesian model comparison, in the context of laminar architectures and the source reconstruction problem in MEG. These arguments apply to the neuronal model, the haemodynamic model and the model of neurovascular coupling that links the two. We hope that this process of refining and improving models will be facilitated by the modelling framework described above.

## Disclosure statement

The authors have no disclosures or conflict of interest.
